# Ketamine as an Anesthetic for Patients with Acute Brain Injury: A Systematic Review

**DOI:** 10.1007/s12028-020-00975-7

**Published:** 2020-04-23

**Authors:** Mads Christian Tofte Gregers, Søren Mikkelsen, Katrine Prier Lindvig, Anne Craveiro Brøchner

**Affiliations:** 1 The Mobile Emergency Care Unit, Department of Anaesthesiology and Intensive Care, Odense University Hospital, Odense, Denmark; 2 The Prehospital Research Unit, Region of Southern Denmark, Odense University Hospital, Odense, Denmark; 3 Department of Regional Health Research, University of Southern Denmark, Odense, Denmark; 4 Department of Clinical Research, University of Southern Denmark, Odense, Denmark; 5 Department of Anaesthesiology and Intensive Care, Kolding Hospital, 6000 Kolding, Denmark

**Keywords:** Anesthetics i.v., Ketamine, Traumatic brain injury, Systematic review, Intracranial pressure

## Abstract

For years, the use of ketamine as an anesthetic to patients suffering from acute brain injury has been debated because of its possible deleterious effects on the cerebral circulation and thus on the cerebral perfusion. Early studies suggested that ketamine could increase the intracranial pressure thus lowering the cerebral perfusion and hence reduce the oxygen supply to the injured brain. However, more recent studies are less conclusive and might even indicate that patients with acute brain injury could benefit from ketamine sedation. This systematic review summarizes the evidence regarding the use of ketamine in patients suffering from traumatic brain injury. Databases were searched for studies using ketamine in acute brain injury. Outcomes of interest were mortality, intracranial pressure, cerebral perfusion pressure, blood pressure, heart rate, spreading depolarizations, and neurological function. In total 11 studies were included. The overall level of evidence concerning the use of ketamine in brain injury is low. Only two studies found a small increase in intracranial pressure, while two small studies found decreased levels of intracranial pressure following ketamine administration. We found no evidence of harm during ketamine use in patients suffering from acute brain injury.

## Introduction

Ketamine is the best-known non-competitive *N*-methyl-d-aspartate receptor antagonist (NMDA). It was first discovered in 1956, and subsequent animal studies showed promising general anesthetic properties [1]. In 1964, ketamine was introduced as an anesthetic for humans and has thus been available for more than 50 years. Ketamine exists as two optical isomers: (S)-(+) and (R)-(−)-2-(2-chlorophenyl)-2-(methylamino)cyclohexanone. At present, ketamine is available as S-ketamine, the more potent of the two optical isomers, and as a racemic mixture containing both (S+) and (R−) ketamine.

Along with the antagonistic effect on the NMDA receptor shown in in vitro studies, ketamine has been shown to interact with opioid, monoaminergic, cholinergic, nikotinergic, and muscarinic receptors assigning ketamine a broad range of effects and side effects [2].

Once bound to the NMDA receptor, ketamine induces a state of dissociation. Contrary to most anesthetic agents, ketamine does not induce dilation of the vascular bed and thus does not induce hypotension, nor does it have a negative chronotropic impact on the heart. This combination makes ketamine a potentially attractive drug when anesthetizing patients with hypotension or patients in whom considerations regarding perfusion pressure during anesthesia are prioritized, as is the case with patients with traumatic brain injury (TBI) [2, 3].

Theoretically, ketamine could be beneficial when treating patients with TBI. However, the drug was abandoned in the 1990s due to the suspicion that the drug should have adverse effects on the intracranial pressure [4].

Immediately after the occurrence of a traumatic brain injury numerous changes are initiated in the suffering brain. Some of these changes are altered propagation of the complex electrical activity that takes place on the surface of the brain.

Various kinds of electrical propagations have been described; all based on external measurements of the waves by electroencephalography EEG or internal measurements with appropriate electrodes. One type of such electrical propagations is spreading depolarizations (SD) [5–7]. These are waves of abrupt, near-complete breakdown of neuronal transmembrane ion gradients and are phenomena that have been associated with poor neurological outcome in patients with TBI [8]. Spreading depolarizations occur in relation to local ischemia but are also seen in the hours or days after ischemia as the remaining tissue suffers from lack of energy supply.

Recent evaluation of retrospective clinical data and case studies have indicated that there might be a therapeutic effect of ketamine following brain injury as it is assumed that ketamine suppresses SD following brain injury [9].

The aim of this systematic review was to assess the currently available data in humans to determine whether the use of ketamine is beneficial in the treatment of patients with TBI by assessing the literature regarding ketamine and intracranial pressure, cerebral perfusion pressure, and the effects on spreading depolarizations.

## Methods

### Protocol and Registration

We performed the systematic review in accordance with the Preferred Reporting Items for Systematic-Reviews and Meta-Analyses (PRISMA) guidelines [10]. No changes were made to the protocol after initiation of the project. The protocol is unpublished. We used Covidence (an online software) [11], to screen titles, abstracts, and full texts.

### Eligibility Criteria

In this systematic review, only human studies were included. The Population, Intervention, Comparator, Outcome (PICO) model [12] applied was defined as: Among adults and children suffering from severe brain injury on ischemic or traumatic basis in a prehospital setting or in an intensive care setting (population), does use of intravenous ketamine during sedation (intervention), compared to no use of ketamine (comparator), affect the intracranial pressure, cerebral perfusion pressure, spreading depolarizations, mortality, or similar hemodynamically variables [heart rate (HR), systolic blood pressure, mean arterial pressure (MAP)] (outcome). We allowed for papers reporting on combined groups of patients with traumatic injury and patients with anoxic damage caused by non-traumatic injury to be included due to the relative scarcity of data on neurologic injury.

We attempted to identify all relevant randomized clinical trials, prospective trials, and retrospective trials. No studies were excluded because of age of publication. Systematic reviews, abstracts, letters, case reports, or unpublished data were not included, though reference lists of systematic reviews were screened for relevant studies not found in the systematic search. Studies focusing on the effect of ketamine on intracerebral hemodynamics in patients suffering only anoxic damage without TBI, or studies focusing on ketamine administrated during other procedures (e.g., abdominal surgery, heart surgery, known intracranial illness, or similar) were excluded.

### Search Strategy

To identify all trials for inclusion, a detailed systematic search the following electronic registers were used: Cochrane Central Register of Controlled Trials, MEDLINE, PubMed, EMBASE, and Google Scholar. The search was performed in May 2019 using the following MESH terms: Brain injury, ketamin, ketamine, or ketalar. The specific search string used was: (((brain injury) OR brain injuries)) AND (((ketamin) OR ketamine) OR ketalar) NOT (animals [mh] NOT humans [mh]). A manual search for additional reports in reference lists of identified studies was performed. Further, ClinicalTrials.gov was examined for ongoing unpublished studies.

### Study Selection

**Fig. 1:**
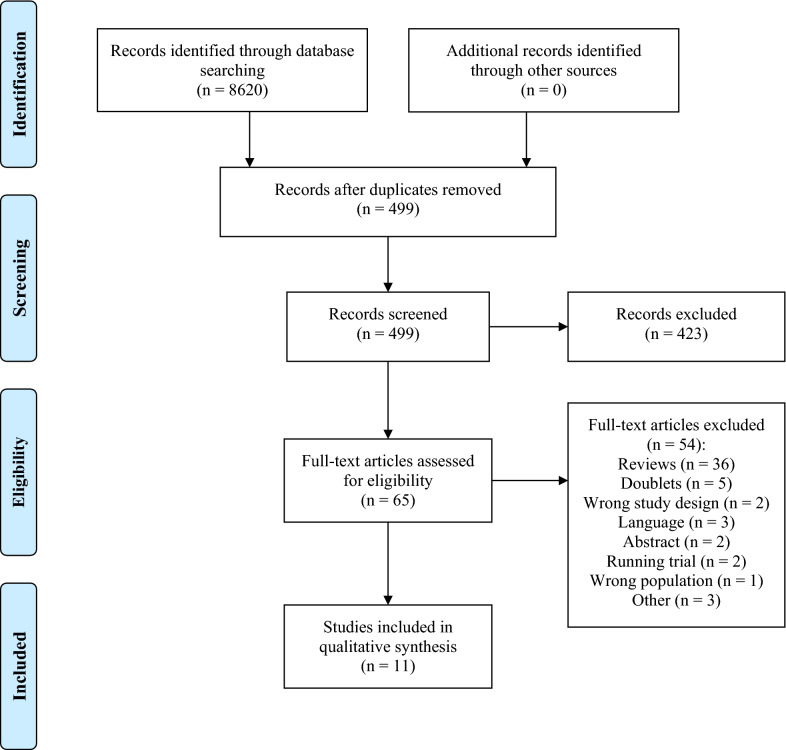
PRISMA flowchart covering the article processing

Two authors (MCTG and ACB) independently screened all titles and abstracts identified in the search and excluded articles not meeting the inclusion criteria. Two authors (MCTG and ACB) screened all full-text papers selected through the first screening. Any disagreement was resolved by discussion between the two authors and with final decision by a third author (SM and/or KL). If any data or other relevant information were missing, the corresponding authors were contacted. None of the investigators were blinded to the publishing journal, authors, or affiliations. A PRISMA flowchart covering the screening process was made, see Fig. 1.

### Validity Assessment

Two investigators (MCTG and ACB) assessed the risk of bias in the included studies. All studies, both randomized, non-randomized, and retrospective studies were evaluated with the standardized Cochrane risk of bias tool at six different domains [13]. Any disagreement was resolved by discussion between the two investigators, and continued disagreements were settled in cooperation with a third investigator (SM).

## Results

### Identification of Studies

The literature searches identified 620 studies to be screened. After removal of duplicates, 499 studies were screened by abstract. A total of 65 studies that investigated the use of ketamine in humans were screened by reading the full text. Eleven studies were eligible for inclusion, containing a total of 334 patients. The reference lists in the included studies were manually screened but did not contribute with any further studies. A frequent cause of exclusion was the use of ketamine on a non-comparable study population (e.g., elective surgery patients, abdominal surgery patients, or patients undergoing cardiac surgery). One study by Carlson et al. [14] was excluded since the study is an evaluation of an already included study by Hertle et al. [9].

### Quality of Included Studies

All included studies were evaluated by design. Overall, the studies showed a large variation in quality, and we found a large degree of heterogeneity in methods and design. One small blinded randomized controlled trial with low risk of selection bias, performance bias, detection bias, and attrition bias was found [15]. However, in that particular study, the risk of reporting bias was unclear. Most of the remaining included studies were prospective studies without randomization and blinding, resulting in a high risk of selection bias, performance bias, and assessment bias [16–20]. Three studies [21–23] used randomized controlled designs but did not have a proper allocation concealment and no blinding, resulting in high risk of bias. Two studies [9, 24] were performed retrospectively with no possibility of randomization and blinding. For further information, see Table 1.

**Table 1:**
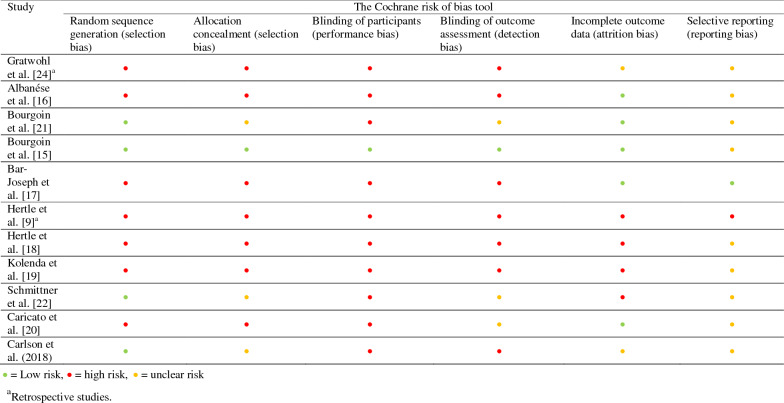
Risk of bias assessment using the Cochrane risk of bias tool

## Main Results

### Human Cerebral Circulation: Intracranial Pressure

Relevant study characteristics from the included studies are presented in Table 2.

**Table 2:** Study characteristics

Study	*N* (of which controls)	Type of brain injury	Age (of patients included)	Intervention	Comparison	Outcome	ICP	CPP	MAP & HR	Evidence of harm
Albanèse et al. [16]	8	Cerebral contusion (TBI)	> 17	*Boli of Ketamine* 1.5, 3, and 5 mg/kg in 6-h intervals	Baseline values of ICP	Mean ICP at 2, 5, 20, and 30 min Mean CPP at 2, 5, 20, and 30 min	Decrease of 1–5 mmHg after 2 min in all groups; increase 3–4 mmHg in two groups after 30 min	No significant differences	No difference between the groups	No
Bar-Joseph et al. [17]	30	Uncontrollable intracranial hypertension (due to illness or trauma)	1–16	*One ketamine bolus* 1–1.5 mg/kg	Baseline values of ICP	ICP at 1 and 2 min before and every min for 10 min after bolus CPP as above	Decrease of 7.8 mmHg after 2 min	Increase of 3.9 mmHg	MAP both seen significantly slightly increased (number unreported) and decreased from 79 ± 11 to 75 ± 11 mmHg in two different groups. HR unreported	No
Bourgoin et al. [15]^a^	25 (13)	TBI (GCS < 8)	17–75	*Ketamine infusion* average of 4.92 ± 1,5 mg/kg/h) for 4 days	*Sufentanil infusion* average of 0.48 ± 0.12 µg/kg/h for 4 days	Mean daily ICP Mean daily CPP	No difference between the groups	No difference between the groups	No difference in MAP or HR	No
Bourgoin et al. [21]^a^	30 (15)	TBI (GCS < 9)	18–75	*Ketamine infusion* 5.7 mg/kg/h with doubling of dose for 15 min, 24 h after onset of sedation	*Sufentanil infusion* 0.42 μg/kg/h with doubling of dose for 15 min, 24 h after onset of sedation	Mean ICP during 15 min Mean CPP during 15 min	No difference between the groups	No difference between the groups	No difference in MAP. HR not reported	No
Caricato et al. [20]	21	TBI (GCS < 8)	18–75	*Ketamine infusion* 6 mg/kg/h for 10 min during suction if cough was present	Baseline values of ICP	Mean ICP Mean C½PP	Increase of 4.1 mmHg during suctioning but unchanged during sedation only	No difference between the groups	No difference in MAP and HR	No
Kolenda et al. [19]	24 (12)	Head injured patients	16–62	*Ketamine infusion* 4.3 mg/kg/h for 10 days	*Fentanyl infusion* 4.2 μg/kg/h for 10 days	Mean ICP once a day Mean CPP once a day	Significant increase in ICP on day 8 (5 mmHg difference) and 10 (8 mmHg difference) but otherwise no difference between the groups	No difference between the groups	Increased MAP of 10 mmHg (significant on day 3 and 7) and HR of 20 bpm (significant on day 2, 3, and 7)	No
Schmittner et al. [22]^a^	24 (12)	TBI with GCS ≤ 8 or SAH with Hunt & Hess > II	20–77	*Ketamine bolus and infusion* 0.5 mg/kg continued infusion up to 2 mg/kg/h for 5 days	*Fentanyl bolus and infusion* 3 µg/kg and continued infusion up to 10 μg/kg/ for 5 days	Mean ICP once a day Mean CPP once a day	No difference between the groups	No difference between the groups	MAP included but unreported. HR not reported.	No
Grathwohl et al. [24]^b^	93 (46)	Combat related TBI	> 18	*Ketamine infusion* 0.3–1.2 mg/kg/h	*Propofol infusion* of 4.5–9 mg/kg/h combined with either remifentanil 0.05–0.3 μg/kg/h, sufentanil 0.001–0.002 μg/kg/h or fentanyl 5–20 μg/kg/h	Ketamine was not associated with death or good neurological outcome. No change in systolic blood pressure was observed	–	–	MAP and HR not reported	No

^a^RCT studies

^b^Subgroup analysis

Of the 11 included studies, seven evaluated the effect on intracranial pressure (ICP) [15–17, 19–22]. All seven studies were conducted in adult [15, 16, 19–22] or pediatric [17] patients with TBI, subarachnoid hemorrhage (SAH), or other intracranial illnesses (e.g., drowned patients with cerebral edema) in an ICU setting and under controlled ventilation. Three studies found no overall difference in ICP between patients receiving ketamine and the matching control group [15, 21, 22]. One study reported slightly elevated ICP of 5 and 8 mmHg in day 8 and 10 with an overall median of 14.6 mmHg [19]. One study found an elevated ICP during tracheal suctioning of head injured patients [20]. The ICP was elevated 4.1 mmHg and 7.5 mmHg in the intervention and control, respectively. Furthermore, it was found that ketamine in this study prevented cough reflexes [20]. One study reported both decreased ICP values (range 1–5 mmHg) 2 min after ketamine administration and elevated (range 3–4 mmHg) ICP values 30 min after ketamine bolus. However, no evidence of harm was reported despite the ICP elevation [16]. One study found decreased ICP values from 25.8 ± 8.4 to 18.0 ± 8.5 mmHg [17] for 2 min following ketamine administration in a pediatric population. In this study, 20 of the 30 included patients received hyperosmolar therapy (either mannitol or 3% NaCl) shortly before ketamine administration [17]. Overall, none of the seven studies reported persistent adverse effects in relation to increased ICP or increased mortality after ketamine administration in brain-injured patients [15, 16, 19–22].

### Human Cerebral Circulation: Cerebral Perfusion Pressure

Seven [15–17, 19–22] studies included monitoring of the cerebral perfusion pressure (CPP). Only one [17] of the seven studies found increased CPP values following ketamine administration. The CPP increased by 3.9 mmHg 2 min after ketamine administration [17].

### Human Cerebral Circulation: MAP and HR

Eight studies [15–17, 19–22, 24] evaluated human cerebral circulation. However, six did not report or did not find any difference in MAP and HR [15, 16, 20–22, 24]. One study found significantly elevated MAP in one group as a response to ketamine administration prior to a potentially distressing intervention, but the actual number was not reported [17]. In the same study, a decreased MAP of 5 ± 11 mmHg was seen when ketamine was administered with the purpose of lowering ICP [17]. However, HR was unreported. One last study saw increased MAP of 10 mmHg on day 3 and 7 and elevated HR with 20 bpm on day 2, 3, and 7 [19].

### Spreading Depolarizations and Burst Suppressions During Ketamine Treatment

Four studies [9, 16, 18, 23] investigated spreading depolarizations (SD) and burst suppressions [electroencephalographic (EEG) activity] during ketamine administrations in brain-injured patients. Relevant study characteristics are presented in Table 3.

**Table 3:** Study characteristics

Study	N	Patients	Age	Intervention	Comparison	Outcome	SD	Evidence of harm
Hertle et al. [9]	26	Brain injury requiring craniotomy	18–79	*Ketamine infusion* 200 mg (median)/h during 2168 h of ECG recordings	Baseline ECG prior to ketamine administration	Number of SD Number of SD clusters	Odds ratio for SD occurrence reduced to 0.38 when receiving ketamine Odds ratio for SD cluster occurrence reduced to 0.2 when receiving ketamine	No
Hertle et al. [18]	43	Brain injury requiring craniotomy	> 50	*Ketamine infusion* (5–250 mg/h)	Baseline ECG prior to ketamine administration	Number of SD Beta frequency activity	Reduced number of SD Increased beta frequency activity	No
Carlson et al. [23]^a^	10	TBI and SAH	> 45	*Ketamine infusion* 0.1 mg/kg/h as basal infusion, titrated after Riker sedation–agitation scale score of 4	Baseline ECG prior to ketamine administration	Number of SD	Reduced number of SD	No

^*a*^RCT study

Two studies evaluated SDs in patients who were treated with ketamine following acute brain injury [9, 18]. Both studies were from the same research group. In one of the two studies, ketamine was found to significantly decrease SD with a dose–response relationship [9].

The other study, a retrospective multicenter study [18], investigated electrocorticographic (ECoG) alpha, beta, delta, and theta frequencies. Alpha, beta, delta, and theta frequencies are direct recordings of electrical potentials associated with brain activity. It was found that in 43 patients, the occurrence of alpha, beta, delta, and theta frequencies, and their mutual proportion was a potential predictor of the occurrence of SD. Firstly, the investigators found that when the occurrence of beta frequencies diminished, an increased number of SDs took place, indicating an association between beta frequencies and SD [18]. Secondly, an increased occurrence of beta frequencies and thus a reduced occurrence of SD were observed, accordingly indicating that ketamine increases the occurrence of beta frequencies and therefore limits SD [18]. With regard to the three remaining electrocorticographic frequencies alpha, delta, and theta, no association was found in relation to the occurrence of SD [18]. This is supported by a third study [16] which found a dose-related burst suppression (a low amplitude fast activity with electrogenic depression) on EEG of patients treated with ketamine. However, these EEG recordings were performed externally as usual, with electrodes on the scalp in contrast to the invasive SD recordings. A fourth small randomized study [23] also demonstrated an inhibitory dose-dependent effect of ketamine on the occurrence of SD with an odds ratio of 13.84 (95% CI 1.99–1000), for the occurrence of SD when not treated with ketamine or when treated with a dose less than 1.15 mg/kg/h.

### Ketamine Dosage

The ketamine dosage applied varied between study groups. The dose used in bolus-only studies ranged from 1 to 5 mg/kg ketamine [16, 17, 22]. In the remaining studies, ketamine was administered by infusion with an infusion rate that varied from 0.3 to 200 mg/kg/h [9, 15, 18–24]. Only four studies [15, 20–22] reported arguments for why the exact ketamine dose was chosen. Typically, the administration was titrated according to the level of sedation, e.g., the Ramsay score or the Riker sedation–agitation scale.

No authors reported potentially fatal or fatal side effects relating to the use of ketamine.

## Discussion

The interest in ketamine has lasted over 50 years, and ketamine has been used for several purposes. Judging by the number of studies, the interest in ketamine as an alternative anesthetic and sedation agent has been constant; only interrupted by a 5-year period in the 1990s where data from the 1970 received renewed interest leading to the conception that ketamine was the main reason for elevated ICP in patients with TBI. In this review, we included 11 studies with a total of 334 patients. Overall, we found no evidence indicating that the use of ketamine in patients with acute brain injury results in worsened cerebral conditions. Specifically, no negative effects were found in ICP, CPP, the occurrence of additional SDs, altered hemodynamics, or mortality. Furthermore, no studies reported any adverse events. These effects, however, cannot be ruled out although no apparent sentinel events were reported.

The overall quality of evidence was low in randomized controlled trials (with only two studies addressing selection bias, performance bias, detection bias, and attrition bias) and very low for observational and retrospective studies.

### Cerebral Circulation

Ketamine is well known for its ability to stabilize and even stimulate the circulatory system compared to most intravenous anesthetics [3]. Its use in treatment of patients suffering from TBI or other conditions with the risk of elevated ICP have been debated since the 1960s after several studies found that ketamine contributed to an elevation in ICP [25]. In this systematic review, only two prospective studies [19, 20] showed elevated ICP during ketamine use. In one study, this elevation was observed during suctioning that is known to induce a raise in ICP, even among healthy volunteers [20]. The other study found elevated ICP in the ketamine group on two different days (5 mmHg and 8 mmHg on day 8 and 10, respectively) with an overall median ICP of 14.6 mmHg [19]. However, this minor elevation of ICP, still within the normal range, does not seem clinically relevant. None of the three randomized controlled trials in adults [15, 21, 22] found increased ICP levels during intravenous infusion of ketamine, nor was any effect on CPP or MAP reported. None of the prospective clinical controlled studies in adults found any change in MAP or CPP during ketamine administration. One randomized controlled trial involving children found a small increase in CPP [17]. Two studies in adults and children [16, 17] found decreased ICP values during ketamine administration. Both used a single bolus of ketamine and observed a decreased ICP shortly after administration.

### Human Cerebral Circulation: MAP and HR

In one study, it was reported that MAP was elevated with 10 mmHg in the ketamine group and the HR was elevated with 20 bpm on day 2, 3, and 7 [19]. The same group saw more stable vital parameters and used less catecholamines in the ketamine group. One other study found both increased and decreased MAP values but did not report HR in the article [17]. Most of the studies used concomitant drugs, e.g., fentanyl, midazolam, or propofol which are known to cause hypotension and mask the sympathetic response of ketamine.

### Spreading Depolarizations During Ketamine Treatment

Four studies [9, 16, 18, 23] included analysis of SDs on ECG recordings. Three studies [9, 16, 23] showed a significant reduction in the occurrence of SD with a ketamine dose-related correlation. Increased SD was associated with poorer neurological outcome [9]. This indicates that ketamine might act as a neuroprotective agent through modulation of SD in the traumatic brain. One study [18] investigated whether spreading depolarizations could be predicted through electrocorticographic frequencies in patients suffering TBI. This study revealed frequent SDs in relation to suppressions of ECoG beta frequencies indicating that this could predict the occurrence of SD. Further, ketamine was found partially to increase the amount of beta frequencies and thereby inhibit the occurrence of SD [18]. However, this was a retrospective study with only a partial effect of ketamine on beta frequencies and SD, indicating that further prospective studies are warranted. Even externally EEG recordings showed burst suppression following ketamine administration supporting the theory of an inhibitory effect on neuronal activity of ketamine [16].

### Controlled Ventilation and Arterial CO_2_

For several years, it has been recognized that controlled ventilation plays an important role in the management of patients with severe TBI [26]. However, one randomized controlled trial and two prospective trials on the effects of ketamine on neurological outcome did not report the targeted partial pressure of CO_2_ in arterial gasses of the patients included in the studies [17, 19, 22]. Furthermore, none of the eleven studies reported how often the partial pressure of CO_2_ was controlled. Two [17, 19] of the three studies [17, 19, 22] that did not control PaCO_2_ found either lowered or elevated ICP levels. All studies used ventilators. No patients were on spontaneous breathing which, in an animal model, has shown to increase intracranial pressure during ketamine sedation [27]. We thus recommend hesitation in relation to conclusions regarding potential correlations between ketamine administration and changes in ICP levels. Non-standardized mechanical ventilation without control of arterial blood gases most probably may contribute to bias and confounding.

### Ketamine Dosage and Timing

The general ketamine dosage varied greatly between the studies. Several studies used concomitant medication (propofol, fentanyl, sufentanil, midazolam, morphine, and etomidate), which might mask the true effect of ketamine. Propofol is widely used as supplemental medication and has been found to lower ICP briefly in patients with brain injury [28].

Furthermore, timing of administration of ketamine appears to be a turning point in animal studies and several treatment regimens have been investigated; administration before experimental TBI, administration simultaneously with TBI, and delayed administration of ketamine [29].

### Ketamine and Children

Only one study investigating the effect of ketamine in children was identified [17]. In this study, the authors showed decreased ICP values and increased CPP shortly after ketamine administration [17].

However, as in many of the other available studies, no information with respect to mechanical ventilator settings or arterial blood gases was provided. Furthermore, two thirds of the patients received hyperosmolar fluid therapy shortly before ketamine administration thus making it difficult to assess the true effect of ketamine [17]. Many attempts have been made in order to elucidate whether anesthetic agents in general affect the developing human brain. Recently, a systemic review [30] investigating the effect of general anesthesia on children has been published. Children exposed to general anesthesia were investigated with respect to various parameters including cognition, sensory-motor development, academic achievement, and neuropsychological diagnoses in later life. Also, magnetic resonance imaging, serum biomarkers, mortality, neurological examination, measurement of head circumference, and impairment of vision were investigated. The studies concerned anesthesia in general, but no specific consideration was made regarding the kind of anesthetic agents utilized. To our knowledge, no studies, neither prospective nor retrospective, have been made on long-term outcome for children treated with ketamine as an anesthetic agent.

Based on the available data in pediatric patients, administration of ketamine to children should be approached with caution as limited data are present.

### Quality of Studies

The overall quality of the included studies varies. Only one study [15] was assessed as having low risk of selection bias, performance bias, detection bias, and attrition bias with unclear risk of reporting bias using the Cochrane risk of bias tool. The general problem in this field is the lack of strong randomized controlled studies. We identified only three minor randomized controlled studies.

To summarize, we conclude that despite over 50 years of research in the field, the amount and quality of data supporting recommendation of the use of ketamine or not in patients with acute brain injury is not impressive. Numerous retrospective and prospective studies have been conducted, but all studies have weaknesses that render a solid recommendation impossible. However, none of the included studies showed any evidence of harm using ketamine to patients suffering from acute brain injury. The feasible and obvious next step would be to design a large controlled randomized double-blinded multicenter study investigating the use of ketamine in patients with acute brain injury addressing multiple confounders.
